# Photochemical α-Cleavage Reaction of 3’,5’-Dimethoxybenzoin: A Combined Time-Resolved Spectroscopy and Computational Chemistry Study

**DOI:** 10.3390/molecules25153548

**Published:** 2020-08-03

**Authors:** Yuanchun Li, Xiting Zhang, Zhiping Yan, Lili Du, Wenjian Tang, David Lee Phillips

**Affiliations:** 1 Department of Chemistry, The University of Hong Kong, Hong Kong 999077, China; fionalyc@connect.hku.hk (Y.L.); heeteen@hku.hk (X.Z.); yzpmc@connect.hku.hk (Z.Y.); 2 Key Laboratory of Flexible Electronics & Institute of Advanced Materials, Jiangsu National Synergetic Innovation Center for Advanced Materials, Nanjing Tech University, Nanjing 211816, China; 3 School of Life Sciences, Jiangsu University, Zhenjiang 212013, China; 4 School of Pharmacy, Anhui Medical University, Hefei 230032, China

**Keywords:** photoinitiator, benzoin, α-cleavage, transient absorption spectroscopy, DFT calculation

## Abstract

Benzoin is one of the most commonly used photoinitiators to induce free radical polymerization. Here, improved benzoin properties could be accomplished by the introduction of two methoxy substituents, leading to the formation of 3’,5’-dimethoxybenzoin (DMB) which has a higher photo-cleavage quantum yield (0.54) than benzoin (0.35). To elucidate the underlying reaction mechanisms of DMB and obtain direct information of the transient species involved, femtosecond transient absorption (fs-TA) and nanosecond transient absorption (ns-TA) spectroscopic experiments in conjunction with density functional theory/time-dependent density functional theory (DFT/TD-DFT) calculations were performed. It was found that the photo-induced α-cleavage (Norrish Type I reaction) of DMB occurred from the nπ* triplet state after a rapid intersystem crossing (ISC) process (7.6 ps), leading to the generation of phenyl radicals on the picosecond time scale. Compared with Benzoin, DMB possesses two methoxy groups which are able to stabilize the alcohol radical and thus result in a stronger driving force for cleavage and a higher quantum yield of photodissociation. Two stable conformations (*cis*-DMB and *trans*-DMB) at ground state were found via DFT calculations. The influence of the intramolecular hydrogen bond on the α-cleavage of DMB was elaborated.

## 1. Introduction

Recently, photoinitiated polymerization has received enormous scientific interest, because it is the basis of a large number of applications in not only traditional areas, such as microelectronics, printing plates, adhesives, photo-curable coatings, inks etc. [1,2,3], but also in high-tech fields including biomaterials, lithography, dental restorative materials, optoelectronics, surface modification and nanotechnology [4,5,6,7,8]. Compared with traditional thermal polymerization, photoinitiated polymerization processes can have many distinct advantages: using a mild reaction temperature instead of employing a high temperature that is required in thermal polymerization; typically providing a lower output of by-products (i.e., chain transfer products); having an unique practical convenience when polymerization is applied in curing of surfaces, especially in cases where fine structures need to be cured but the curing material (such as dental fillers) is located at some places where heating is not allowed [9].

**Figure 1:**
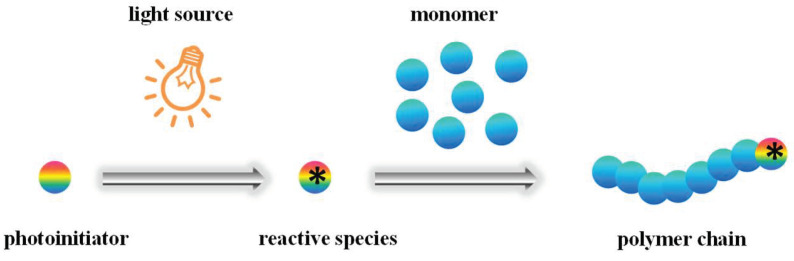
Schematic representation of photo-induced polymerization.

It is noted that light only plays a prominent role in the beginning step of the photo-induced polymerization process (Figure 1), namely light absorption and generation of initiating species from photoinitiators [9]. The photoinitiator is a molecule or a group of molecules that creates reactive species such as radicals, cations and in rare cases, anions and weak bases [1,10,11], which are able to initiate polymerization upon photoexcitation (UV or visible light). They are vital for the polymerization process, especially for reaction rate and efficiency [12]. Therefore, there has also been interest in studying the photochemistry of photoinitiators to determine the nature of the reactive species and acquire related dynamical information via ultrafast transient absorption spectroscopy, real-time IR spectroscopy, electron spin resonance spectroscopy and so on. [6,13,14].

**Scheme 1:**
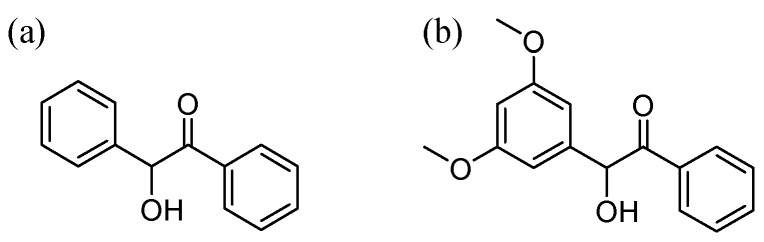
Structure of (**a**) benzoin and (**b**) DMB (3’,5’-dimethoxybenzoin).

Generally, photoinitiators for free radical polymerization can be divided into two categories according to the pathways of generating the radicals: (1) photodissociation type and (2) H-abstraction type [15]. Most photodissociation type photoinitiators are aromatic carbonyl compounds, and benzoin (Scheme 1) is a typical representative among them [15,16,17]. It is not only one of the most commonly used photoinitiators to induce free radical polymerization of vinyl monomers, but is also widely applied in the polymer industry [1,9]. Nevertheless, there are two major disadvantages of benzoin that limit its further application: the unsatisfactory photodissociation quantum yield and poor light absorption in the near-UV region. Recently, it was reported that Nergis Arsu and co-workers had synthesized a benzoin derivative (MTB) by introducing a methylthio group, which showed higher molar absorptivity and a desired red-shifted absorption compared to benzoin [18]. However, studies that focus on improving the photodissociation quantum yields of benzoin-type photoinitiators are rare.

Herein, we are pleased to report that improved benzoin properties could be found by the introduction of two methoxy substituents at the meta-positions of the benzyl moiety, leading to the formation of 3’,5’-dimethoxybenzoin (DMB) (Scheme 1). It was found that DMB had a higher photo-cleavage quantum yield (0.54) than benzoin (0.35) [19], which suggests that DMB is a more efficient photoinitiator. Taking aim at unravelling the photochemical reaction mechanism of DMB and obtaining direct dynamical information of the transient species involved, femtosecond transient absorption (fs-TA) and nanosecond transient absorption (ns-TA) spectroscopic experiments were performed in acetonitrile (MeCN). DFT and TD-DFT calculations were carried out at the B3LYP/6-311 + g(d,p) (MeCN) level of theory in order to acquire more details from a theoretical perspective and these results were employed to help assign the transient species detected from the time-resolved experiments. The parent molecule benzoin was also studied for comparison purposes using the same methods.

## 2. Results and Discussion

### 2.1. Steady-State Study and Photoproduct Analysis

**Figure 2:**
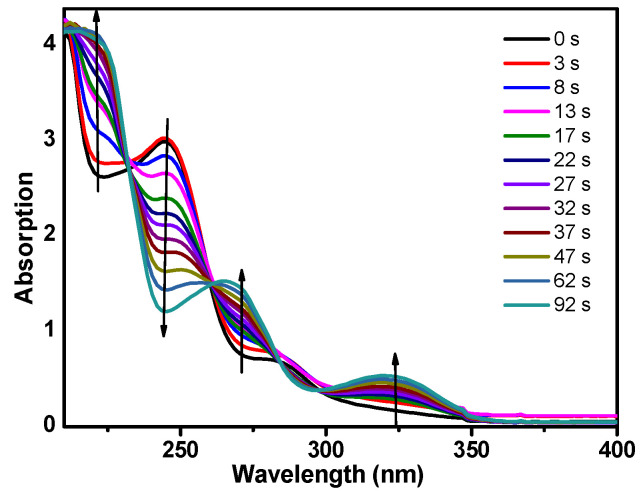
Steady-state absorption spectra change of DMB in MeCN (acetonitrile) with the increase of irradiation time. The wavelength of the irradiation monochromatic light is 266 nm.

Figure 2 displays the steady-state absorption spectra of DMB after different irradiation time intervals by 266 nm monochromatic light. As the irradiation time increases, the DMB absorption spectrum changes into a different spectrum (from 0 to 92 s) and there are five isosbestic points located at ≈ 210, 224, 247, 267 and 300 nm and this suggest the formation of new compounds. The steady-state fluorescence spectrum of DMB in MeCN was also obtained (see Figure S1). The emission band (318 nm) indicates that fluorescence is one of the deactivation pathways of the first singlet excited state of DMB (denoted as DMB (S_1_)).

**Figure 3:**
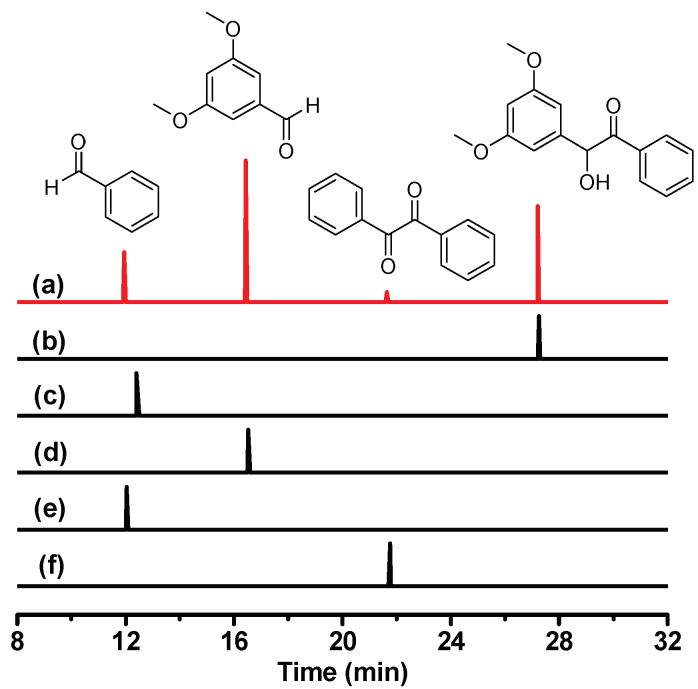
GC (gas chromatography) traces in MeCN of (**a**) DMB after 266 nm irradiation; (**b**) DMB; (**c**) benzoic acid; (**d**) 3,5-dimethoxybenzaldehyde; (**e**) benzaldehyde; (**f**) benzil.

To investigate the photochemical reaction of DMB, photo-product analysis was performed after photolysis of DMB in MeCN. Figure 3a depicts the GC (gas chromatography) trace of DMB in MeCN after irradiation by 266 nm. Four signal peaks at 11.94, 16.44, 21.64 and 27.20 min were observed, which correspond to four different products. Some other photoproducts may exist, but their concentrations are too low to be detected and thus will not be considered further. As it has been reported that the parent molecule benzoin underwent an α-cleavage reaction after photoexcitation [19,20,21], we assume that the photochemical reaction of DMB is also α-cleavage in character. Thus the final photoproducts should include an aryl aldehyde, a benzaldehyde and several radical recombination compounds such as benzil and 1,2-bis(3,5-dimethoxyphenyl)ethane-1,2-diol (the self-quenching product of the dimethoxy-benzyl alcohol radical). Additionally, it is worth mentioning that oxygen dissolved in the solution may oxidize the aldehydes into carboxylic acids as the DMB solution was prepared in an air-equilibrium condition. 

To verify this conjecture, we performed GC experiments for several standard compounds (benzoic acid, 3,5-dimethoxybenzaldehyde, benzaldehyde and benzil) under the same experimental conditions. As shown in Figure 3, it is clear that the main photoproducts of DMB consist of 3,5-dimethoxybenzaldehyde, benzaldehyde and benzil. These products, especially benzil (although the yield is low), provide solid evidence that the photochemical reaction of DMB is an α-cleavage accompanied by the generation of radical species. The photo-cleavage quantum yield of DMB (0.54) is remarkably higher than that of benzoin (0.35) [19], which makes DMB attractive to be studied for its great potential in applications as an efficient photoinitiator.

Furthermore, ^1^H-NMR spectra were used to detect the photolysis reactions of DMB in CD_3_CN (see Supplementary Materials). As shown in Figure S2, the chemical shifts of aryl aldehydes appears after irradiation for 3 min (*δ* = 9.90, 10.01). When the irradiation time increases to 30 min, the chemical shifts of α-H of DMB (*δ* = 4.44) almost disappears while the chemical shifts of aryl aldehydes increases evidently, which is characteristic of the breaking of an α-bond of the DMB carbonyl into the photoproduct aryl aldehydes.

### 2.2. Time-Resolved Transient Absorption Spectroscopy

**Figure 4:**
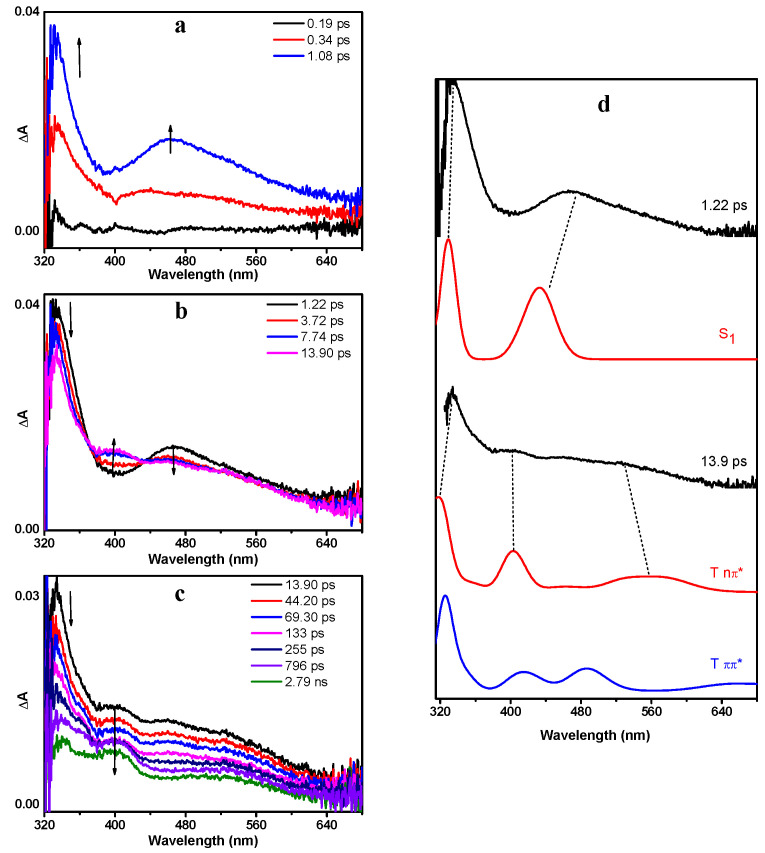
The fs-TA (femtosecond transient absorption) spectra of DMB in MeCN acquired after 266 nm irradiation (**a**) from 0.19 ps to 1.08 ps, (**b**) from 1.22 ps to 13.9 ps, (**c**) from 13.9 ps to 2.79 ns; (**d**) Comparison between the calculated absorption spectra of DMB (S_1_), DMB (T nπ*), DMB (T ππ*) and the fs-TA spectra recorded at 1.22 ps and 13.9 ps. The calculations were performed at the TD-B3LYP/6-311 + g(d,p) (MeCN) level of theory. The scale factor is 1.05 (applied to energy (*x*-axis)) and the half-width is 1000 cm^−1^.

To obtain further insight into the reaction mechanism(s) and gain direct information of the transient species involved, fs-TA and ns-TA experiments were conducted in MeCN. The same measurements were also carried out for benzoin for comparison purposes. Figure 4 presents the fs-TA spectra of DMB in MeCN after 266 nm irradiation. As shown in Figure 4a, DMB was first excited to the S_n_ state. Then, it relaxed to the S_1_ state rapidly via internal conversion, which absorbed at 330 nm and 470 nm. The simulated UV—vis spectrum of DMB (S_1_) by TD-B3LYP calculations exhibits excellent similarities with the corresponding experimental spectrum (Figure 4d), which provides convincing evidence for this assignment.

Subsequently, accompanied by the decay of DMB (S_1_), a new band centered at 400 nm emerged (Figure 4b). The observation of two isosbestic points at 372 nm and 435 nm indicates that DMB (S_1_) underwent a dynamical transformation to another species (denoted as X_400_) from 1.22 to 13.9 ps. This transformation is proposed to be an ISC process based on its time scale, and thus species X_400_ should be triplet state in nature. According to previous reports, it has been revealed that benzoin populates the nπ* triplet state after photoexcitation and efficient ISC [19]. As an analogue of benzoin, it is reasonable to speculate that DMB also generates a nπ* triplet state species after irradiation. In order to verify this hypothesis, we carried out TD-DFT calculations. As expected, the fs-TA spectrum at 13.9 ps exhibits considerable resemblance with the computed absorption spectra of DMB (T nπ*), but displays great difference with the spectra of DMB (T ππ*) (Figure 4d). Hence, the new species X_400_ can be reasonably assigned to DMB (T nπ*). Furthermore, the energies of selected excited singlet and triplet states relative to the singlet ground state of DMB are summarized in Table S2. It can be found that the energy of S_1_ (3.316 eV) is very close to T_3_ (3.291 eV), suggesting that this rapid ISC process occurs between the S_1_ and T_3_ states of DMB. 

**Figure 5:**
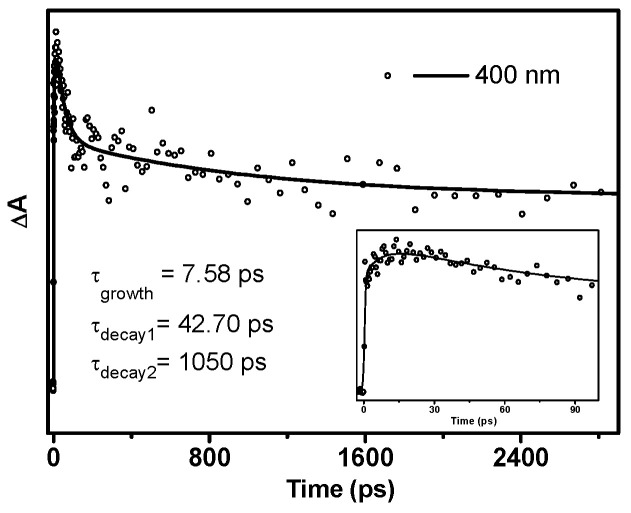
Kinetics of the absorption band at 400 nm acquired by fs-TA experiments of DMB in MeCN. (Inset) The enlargement of the kinetics from 0 to 100 ps. The solid line depicts the best fit to the data.

From 13.9 ps, DMB (T nπ*) started to decay without forming an obvious new species (Figure 4c). The transient absorption signal at 400 nm can be well-fitted by a three-exponential function with one rise time constant and two decay time constants (Figure 5). The 7.6 ps rise component corresponds to the time constant of ISC. As the quantum yield of α-cleavage of DMB is high (0.54), this decay channel should be very competitive compared with other deactivation pathways, which means that the time constant of the α-cleavage process is relatively small from the perspective of dynamics. Therefore, the shorter time constant (42.7 ps) is tentatively attributed to the α-cleavage process of DMB (T nπ*), and the longer decay component (1050 ps) may arise from the other deactivation pathways of DMB (T nπ*), such as returning to the ground state via ISC, being quenched by oxygen dissolved in solution and/or possibly other processes.

**Figure 6:**
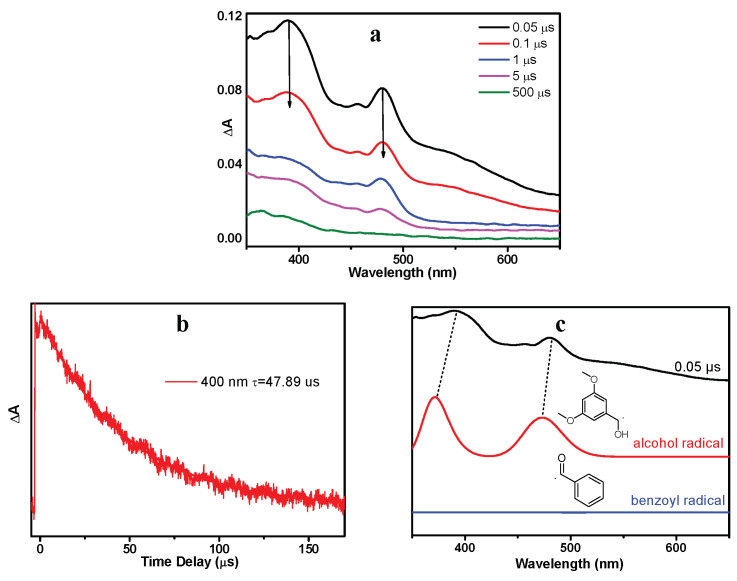
(**a**) ns-TA (nanosecond transient absorption) spectra of DMB in MeCN after 355 nm irradiation. (**b**) Transient decay of DMB in MeCN monitored at 480 nm. (**c**) Comparison of the computed absorption spectra of alcohol radical and benzoyl radical with the ns-TA spectrum recorded at 0.05 μs. The calculations were performed at the TD-B3LYP/6-311 + g(d,p) (MeCN) level of theory. The scale factor is 1.05 (applied to energy (*x*-axis)) and the half-width is 1000 cm^−1^.

**Scheme 2:**
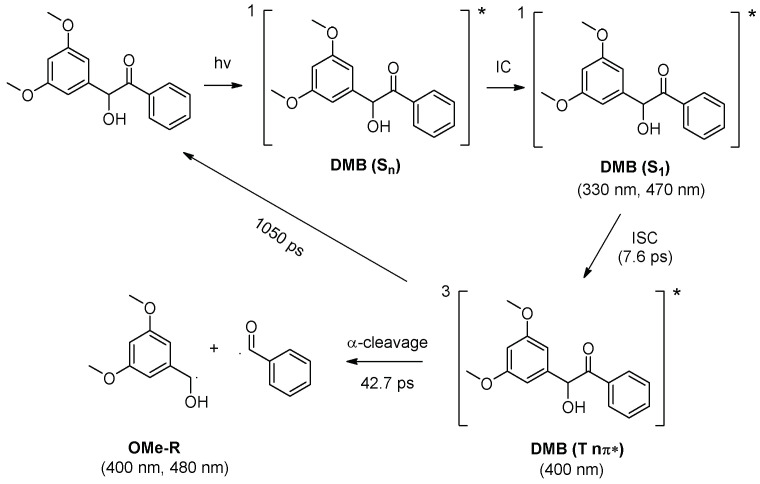
Proposed reaction mechanism of the photochemical α-cleavage reaction of DMB in MeCN. Absorbance for the transient species detected from fs-TA and/or ns-TA spectra are given.

As shown in Figure 6a, a new species with a maximum absorption band at 400 nm and a shoulder band at 480 nm were observed (denoted as X_480_). Species X_480_ was long-lived and its decay time constant was determined to be 47.9 µs (Figure 6b). Figure 6c (red line) presents the simulated absorption spectrum for dimethoxy-benzyl alcohol radical (OMe-R), which exhibits great resemblance with the ns-TA spectrum of DMB in MeCN recorded at 0.05 μs. Therefore, the species X_480_ is assigned to the alcohol radical generated by DMB (T nπ*). Benzoyl radical (Ben-R), the counterpart species of the alcohol radical, is not recognized in the spectra because it does not absorb in this spectral region (Figure 6c blue line), which agrees well with previous literature reports [19]. The proposed reaction mechanism of the photochemical α-cleavage reaction of DMB in MeCN is presented in Scheme 2.

After the alcohol radical and benzoyl radical were fully generated, the stable final products can be produced through the following pathways: (1) Hydrogen abstraction from a hydrogen donor. The predominant photoproducts 3,5-dimethoxybenzaldehyde and benzaldehyde were mainly generated in this way. Because the concentration of the DMB solution prepared for the experiment is very low (<1 × 10^−4^ M), the hydrogen donors are most likely solvent molecules in most cases. However, hydrogen abstraction may also occur between the radicals and the DMB molecules, which is accompanied by the appearance of large ketyl radicals as shown in Scheme S1 [20]. These large radicals are likely to stabilize themselves by dissociation to form aldehydes or combine with each other. However, products of the self-combination of these large radicals were not detected under our experimental conditions. (2) Radical recombination of the alcohol radical and benzoyl radical. These products include DMB, benzil and diole. Moreover, since the experiments were conducted in aerated solution, it is possible that the free radicals can be oxidized by oxygen dissolved in the solution, leading to the formation of benzoic acid and dimethoxy-benzoic acid via peroxide intermediates.

**Figure 7:**
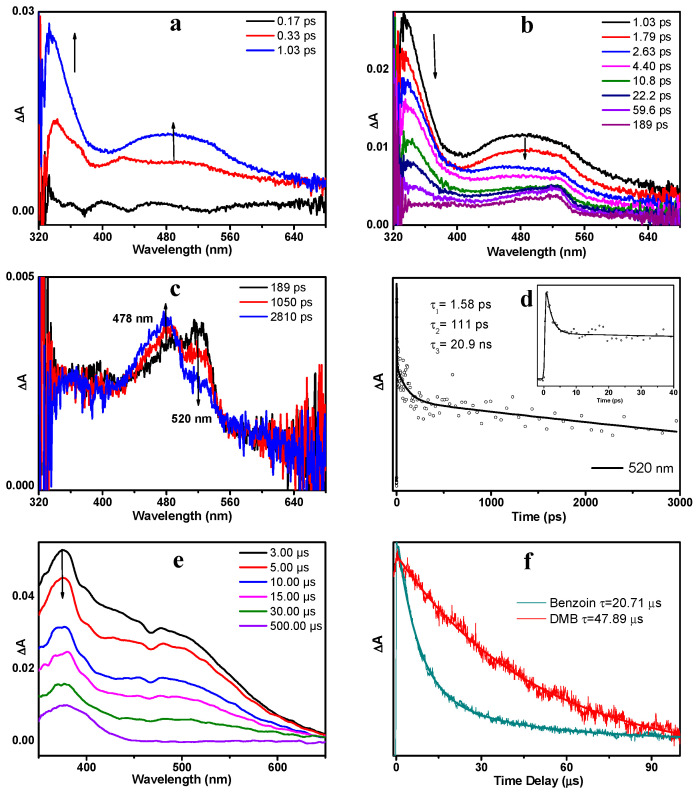
The fs-TA spectra of benzoin in MeCN obtained upon 266 nm excitation (**a**) from 0.17 ps to 1.03 ps, (**b**) from 1.03 ps to 189 ps, (**c**) from 189 ps to 2.81 ns. (**d**) Kinetics trace monitored at 520 nm in MeCN. The solid line depicts the best fitting. (Inset) The enlargement of kinetic trace from 0 to 40 ps. (**e**) ns-TA spectra of benzoin in MeCN obtained upon 266 nm excitation. (**f**) Normalized transient absorption dynamics of benzoin and DMB in MeCN monitored at 400 nm.

To compare and contrast with the photoexcited processes of DMB, the parent molecule benzoin was also studied using same experimental methods, and the results are briefly discussed here. As displayed in Figure 7a, upon 266 nm excitation, benzoin went to the S_n_ state and then relaxed to the S_1_ state via an IC process within 1.1 ps. The absorption bands of benzoin (S_1_) were maximized at 334 and 480 nm. As expected, the early spectra of benzoin resemble those of DMB significantly. As the delay time increased, benzoin (S_1_) decayed accompanied by the growth of a new species that possesses a weak absorption band at 520 nm (Figure 7b). This species is assigned to benzoin (T nπ*) based on similar results observed in previous studies [17,19]. Figure 7d depicts the kinetics trace monitored at 520 nm in MeCN and the decay dynamics can be well-fitted by three exponentials with the time constants of 1.58 ps, 111 ps and 20.9 ns. From 189 ps, the absorption band at 520 nm decreased in intensity while a sharp-pointed band at 478 nm increased in intensity (Figure 7c), which suggests a precursor-successor type of relationship between these two species. Therefore, this spectral variation is attributed to the α-cleavage process of benzoin (T nπ*) and the absorption band at 478 nm is assigned to the radical generated via this α-cleavage. 

The ns-TA spectra of benzoin (Figure 7e) present the slow decay process of the benzyl alcohol radical (BA-R), which has broad absorption bands ranging from 350–650 nm. As expected, the spectral profiles of BA-R and OMe-R display great similarities with each other, except that the shoulder band of OMe-R (478 nm) blue-shifts a little compared with that of BA-R (500 nm). However, the lifetime of BA-R (20.7 µs) is much shorter than that of OMe-R (47.9 µs) (see Figure 7f). To obtain a deeper understanding of this difference, we studied the stability of these radicals. The same benzoyl radicals are formed via α-cleavage reactions of both DMB and benzoin, but the corresponding alcohol radicals are different in structure. It is proposed that the methoxy group in OMe-R has strong electron-donating ability and is able to stabilize the radical. Such stabilization offers a further driving force for α-cleavage, which might rationalize the higher decomposition quantum yield of DMB in comparison to benzoin.

### 2.3. DFT Calculations

**Figure 8:**
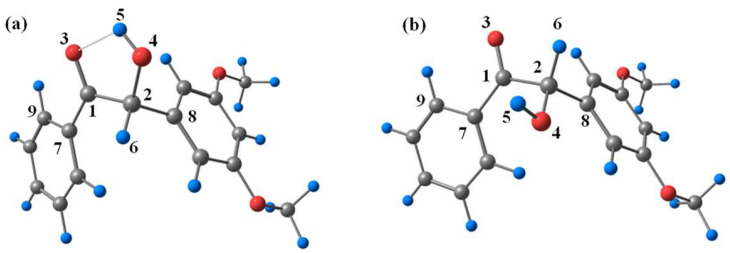
Optimized geometries of (**a**) *cis*-DMB and (**b**) *trans*-DMB at ground state.

To gain insights into the electronic structural properties of DMB, DFT calculations were carried out at the B3LYP/6-311 + g(d,p) (MeCN) level of theory. Similar to benzoin [16], two stable conformations (*cis*-DMB and *trans*-DMB) of DMB in the ground state were found on the potential energy surface (see Figure 8). The computed energies and selected geometrical parameters are summarized in Table S1. *Cis*-DMB lies 3.97 kcal/mol below *trans*-DMB in energy, which is due to the intramolecular hydrogen bond of *cis*-DMB.

The computed frontier orbitals of *cis*-DMB and *trans*-DMB are visualized in Figure S3. It is obvious that both the HOMO and LUMO of *cis*-DMB greatly resemble those of *trans*-DMB, which indicates that the conformation does not have much influence on the electron density distribution of the frontier orbitals. The electron density of the HOMO is mainly localized on the hydroxy-benzyl moiety, while the LUMO shifts to the benzoyl moiety. This HOMO→LUMO transition can be regarded as an excitation into a ππ* singlet excited state with significant charge transfer character. For comparison, the visualization of the computed LUMO and HOMO of *trans*-benzoin is also presented in Figure S3. Surprisingly, different from *trans*-DMB (S_1_), *trans*-benzoin (S_1_) is in the nπ* state, which is consistent with results from previous studies on benzoin [14,17]. We think that such a difference in behavior for can be attributed to the existence of the electron-donating methoxy groups of DMB. It is inferred that these two methoxy groups make the energy levels of the π and π* orbitals of DMB increase while they have little influence on the energy level of the n orbital. Therefore, it is the ππ* state rather than the nπ* state that becomes the lowest singlet excited state for DMB.

**Figure 9:**
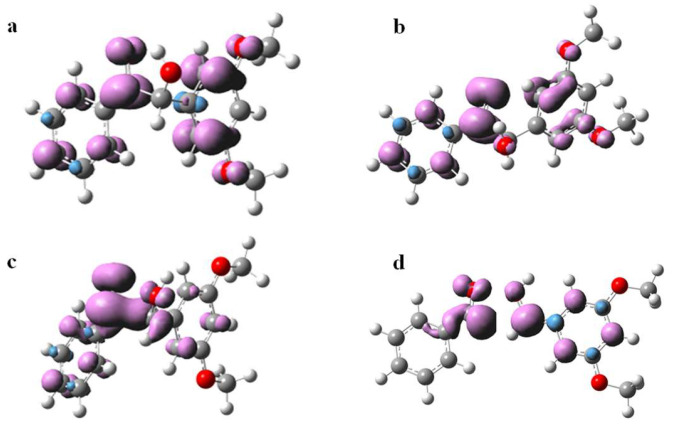
Isosurfaces of the spin density of (**a**) DMB (T ππ*), (**b**) DMB (T nπ*), (**c**) transition state of α-cleavage and (**d**) radicals generated by α-cleavage at the optimized geometries (triplet state). The calculations were carried out at the B3LYP/6-311 + g(d,p) (MeCN) level of theory (Isovalue = 0.005).

The spin density surfaces of DMB (T nπ*) and DMB (T ππ*) were also computed. As displayed in Figure 9, the triplet *cis*-DMB is in a ππ* state while the triplet *trans*-DMB is in a nπ* state, which suggests that the conformation, or in other words intramolecular hydrogen bond, affects the nature of the triplet state significantly. These results are consistent with results observed in some previous studies that found the intramolecular hydrogen bond can increase the ππ* character in the ketone’s triplet state [20]. According to the transient absorption spectroscopy study on DMB in Section 2.2, the nπ* triplet state is more reactive towards α-cleavage than the ππ* triplet state. This phenomenon can be rationalized by the direct coupling of the nπ* state with the highly dissociative nσ* state, while the ππ* state cannot form this coupling [22]. Therefore, *cis*-DMB is not favorable to α-cleavage as the intramolecular H-bond can increase the ππ* character. In addition, due to the intermolecular hydrogen bond between O_3_-H_5_ (see Figure 8a for numbering), a five membered ring (C_1_-C_2_-O_4_-H_5_-O_3_) is formed and the relative positions of the carbonyl moiety and the α-phenyl ring are “locked”, which may slow down the cleavage of the C_1_-C_2_ bond. This interpretation is supported by Wagner and Stratton’s report [23] which showed that the conformation of the phenyl ring in an α-phenylcyclohexanone relative to the carbonyl segment acted as an on/off switch for the α-cleavage in the triplet state.

In summary, the intramolecular hydrogen bond can perhaps inhibit the α-cleavage of DMB through (1) increasing the extent of the ππ* character at the triplet state and (2) ‘fixing’ the conformation that is unfavorable for the C_1_-C_2_ bond cleavage. Furthermore, because polar solvents can competitively break the intramolecular H-bond and increase the concentration of the *trans*-DMB conformation which favors α-cleavage in the equilibrium between *cis*-DMB and *trans*-DMB, it is reasonable to infer that the α-cleavage of DMB (reaction rate and quantum yield) is also greatly affected by solvent effects. A quantitative study of the solvent dependence for DMB utilizing time-resolved transient spectroscopy is planned to be explored in future studies.

Finally, the spin density surfaces of the transition state and product of α-cleavage for DMB in the triplet state are also presented in Figure 9. It is evident that a pair of spin parallel electrons are located on the carbon atoms of the two fragments respectively when the C_1_-C_2_ single bond breaks, confirming that the products of the reaction are two free radicals from a theoretical perspective.

## 3. Materials and Methods 

### 3.1. Materials

The DMB compound was prepared following the procedure previously reported in the literature [24]. The sample of benzoin, benzoic acid, benzaldehyde and benzil were commercially obtained and used as-is. The 3,5-dimethoxybenzaldehyde was synthesized using the photolysis method described in the Supplementary Materials. Spectroscopic grade acetonitrile was employed to prepare the sample solutions which were adjusted to ≈1 absorbance at the excitation wavelength (266 nm or 355 nm) for the fs-TA and ns-TA experiments. 

### 3.2. Experimental and Computational Methods

#### 3.2.1. GC Experiments for Photoproduct Analysis

The photoproduct distributions of DMB were studied by gas chromatography (GC). The gas chromatograph was an Agilent Technologies 7890A. A Hewlett Packard capillary column with 25-m length, 0.32-mm I.D., and 0.52-μm film thickness was used with the following oven temperature program: 70 ℃ for 2 min, 10 ℃ min^−1^ increase until a final temperature of 280 ℃. The injector was set to 280 ℃ and the injection volume was 2 μL. The sample solutions were prepared at concentrations of 5000 ppm.

#### 3.2.2. Measurements of Quantum Yield

The photolysis reaction quantum yield of DMB was determined using the methods described in the Supplementary Materials.

#### 3.2.3. fs-TA and ns-TA Experiments

The fs-TA and ns-TA experiments here utilized the same experimental apparatus and methods that have been detailed in previous work [25,26,27].

#### 3.2.4. Theoretical Calculations

DFT calculations were performed utilizing the B3LYP method [28,29] with a 6-311 + g(d,p) basis set for the computations of the species of interest in their ground state and triplet state. The vibrational frequencies were calculated to determine whether the optimized geometry of interest was a local minima or not. TD-DFT calculations were carried out to optimize the structures in the singlet excited state as well as to compute the UV—vis absorption spectra. The polarizable continuum model (PCM) [30,31] in MeCN on the gas-phase structures was used to examine the solvent polarity effect on the stability of the relevant species. All the calculations reported here were done with the Gaussian 09 program package [32]. 

## 4. Conclusions

In this study, photoproduct analysis, fs-TA and ns-TA spectroscopic experiments in conjunction with theoretical calculations were performed to elucidate the photo-cleavage mechanisms of DMB. Upon 266 nm photoexcitation, DMB (S_0_) went to DMB (S_n_) and then relaxed to DMB (S_1_) via IC. Later, DMB (S_1_) underwent a rapid ISC process in 7.6 ps and generated DMB (T nπ*). Subsequently, the α-cleavage of DMB (T nπ*) led to the formation of a dimethoxy-benzyl alcohol radical and a benzoyl radical on the picosecond time scale. The parent molecule benzoin was also studied for comparison with the results for DMB. The decomposition quantum yield of DMB is higher than that of benzoin, which makes DMB attractive as a more efficient photoinitiator. Such a difference may be due to the methoxy groups of DMB which are able to stabilize the alcohol radical and thus result in a stronger driving force for the cleavage reaction of interest. In the triplet state, *cis*-DMB has a ππ* character which disfavors α-cleavage while *trans*-DMB is in the nπ* state which favors α-cleavage. It is proposed that the intramolecular hydrogen bond can reduce the α-cleavage rate in the triplet state by increasing the extent of ππ* character or ‘fixing’ the *cis*-conformation that is unfavorable to the C_1_-C_2_ bond breaking. Our results are helpful in understanding the α-cleavage reaction mechanism of aromatic carbonyl compounds and will be useful for the design of new benzoin-type photoinitiators with higher efficiency.

## Supplementary Materials

The following are available online: Figure S1: Steady-state emission spectrum of DMB in MeCN at room temperature (λ_ex_ = 266 nm). Figure S2: Change in the ^1^H-NMR spectra of DMB in CD_3_CN at different irradiation time (0, 3, 30 min) with 320 nm monochromatic light. Figure S3: Visualization of the computed LUMO, HOMO and HOMO-1 of cis-DMB, trans-DMB and trans-benzoin at the optimized ground state geometries. Scheme S1: Two possible photochemical reaction mechanisms of DMB. Table S1: Structural parameters of the optimized geometries, total and relative energies, as well as dipole moments of *cis*-DMB and *trans*-DMB. Table S2. Energies of selected excited singlet and triplet states relative to the singlet ground state of *trans*-DMB. Synthesis of photoproduct 3,5-dimethoxybenzaldehyde. Measurements of quantum yield of photolysis reaction of DMB. Cartesian coordinates of the optimized geometries obtained from the DFT or TD-DFT calculations.
