# Supplementary material for: Photochemical α-Cleavage Reaction of 3’,5’-Dimethoxybenzoin: A Combined Time-Resolved Spectroscopy and Computational Chemistry Study
Source: Molecules. 2020 Aug 3;25(15):3548. doi: 10.3390/molecules25153548 (PMC7435414; doi:10.3390/molecules25153548)
Supplement: Supplementary file 1 [file molecules-25-03548-s001.pdf]

Supplementary Materials for

Photochemical α-Cleavage Reaction of 3',5'-Dimethoxybenzoin: A Combined Time-Resolved Spectroscopy and Computational Chemistry Study

Yuanchun Li [1], Xiting Zhang [1], Zhiping Yan [1,4], Lili Du [1,3,*], Wenjian Tang [2,*] and David Lee Phillips [1,*]

[1] Department of Chemistry, The University of Hong Kong, Hong Kong S.A.R., P. R. China; fionalyc@connect.hku.hk (Y.L.); heeteen@hku.hk (X.Z.); yzpmc@hku.hk (Z.Y.);
[2] School of Pharmacy, Anhui Medical University, Hefei, 230032, P. R. China;
[3] School of Life Sciences, Jiangsu University, Zhenjiang, 212013, P. R. China;
[4] Key Laboratory of Flexible Electronics & Institute of Advanced Materials, Jiangsu National Synergetic Innovation Center for Advanced Materials, Nanjing Tech University, Nanjing, 211816, P.R. China;
* Correspondence: ailleen@hku.hk (L.D.); ahmupharm@126.com (W.T.); phillips@hku.hk (D.L.P.); Tel.: +852-6761-4757 (L.D.); +86-13721091768 (W.T.); +852-2859-2160 (D.L.P.)

Table of Contents

Figure S1. Steady-state emission spectrum of DMB in MeCN at room temperature (λ_{ex} = 266 nm)........2

Figure S2. Change in the ^{1}H-NMR spectra of DMB in CD_3CN at different irradiation time (0, 3, 30 min) with irradiation by 320 nm monochromatic light........2

Figure S3. Visualization of the computed LUMO, HOMO and HOMO-1 of *cis*-DMB, *trans*-DMB and *trans*-benzoin at the optimized ground state geometries..........3

Scheme SI. Two possible photochemical reaction mechanisms of DMB. 3

Table S1. Structural parameters of the optimized geometries, total and relative energies, as well as dipole moments of *cis*-DMB and *trans*-DMB. 3

Table S2. Energies of selected excited singlet and triplet states relative to the singlet ground state of trans-DMB........4

Synthesis of photoproduct 3,5-dimethoxybenzaldehyde 4

Measurements of quantum yield of photolysis reaction of DMB........4

Cartesian coordinates and electronic energies of the optimized geometries obtained from the DFT or TD-DFT calculations........5

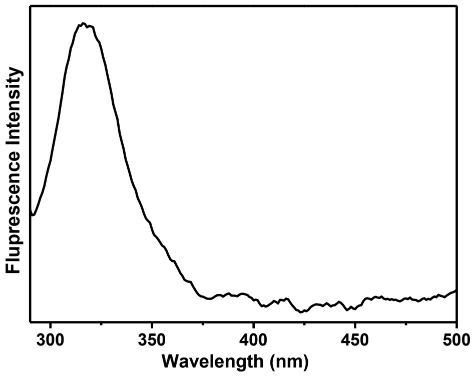

Figure S1. Steady-state emission spectrum of DMB in MeCN at room temperature (λ_{ex} = 266 nm).

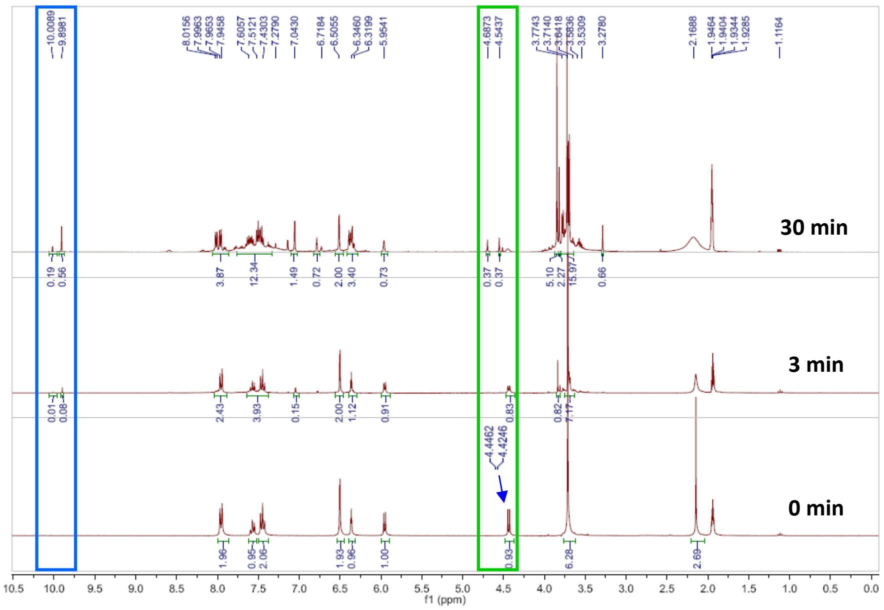

Figure S2. Change in the ^{1}H-NMR spectra of DMB in CD_3CN at different irradiation time (0, 3, 30 min) with irradiation by 320 nm monochromatic light.

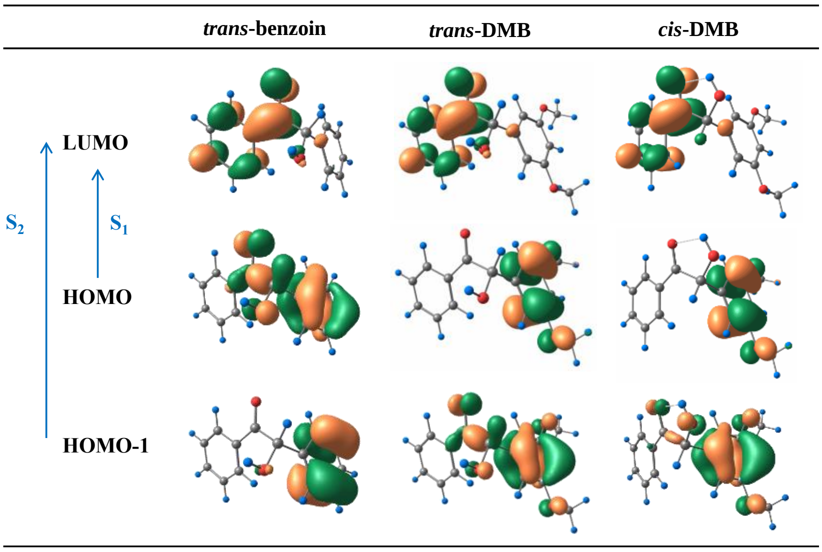

Figure S3. Visualization of the computed LUMO, HOMO and HOMO-1 of *cis*-DMB, *trans*-DMB and *trans*-benzoin at the optimized ground state geometries. The calculation were performed at B3LYP/6-311+g(d,p) (MeCN) level of theory (contour value: 0.0432).

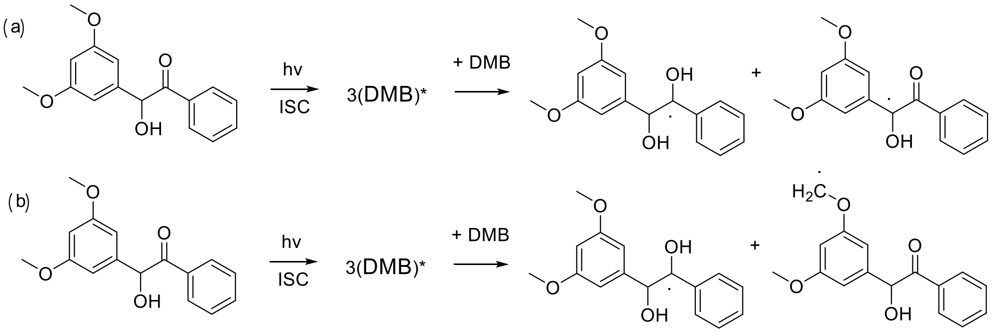

Scheme SI. Two possible photochemical reaction mechanisms of DMB.

Table S1. Structural parameters of the optimized geometries, total and relative energies as well as dipole moments of *cis*-DMB and *trans*-DMB.

Parameters [1]	*cis*-DMB	*trans*-DMB
$r(C_1C_2)$	1.543	1.550
$r(C_1C_7)$	1.526	1.525
$r(C_2C_8)$	1.484	1.492
$r(C_1O_3)$	1.224	1.222
$r(C_2O_4)$	1.420	1.423
$r(O_4H_5)$	0.973	0.964
$r(O_3H_6)$	3.119	2.407
$r(O_3H_5)$	1.963	3.542
$r(O_3O_4)$	2.586	3.471
$\angle C_2O_4H_5$	105.773	108.597

$\angle O_4H_5O_3$	119.592	77.942
$\varphi C_1C_2O_4H_5$	-12.903	-71.671
$\varphi C_2O_4H_5O_3$	9.955	22.517
$\varphi C_8C_2O_4H_5$	109.793	163.428
$\varphi C_9C_7C_1O_3$	11.799	-11.430
Total energy (Hartree)	-920.236	-920.230
Relative energy (kcal/mol)	0	3.97
Dipole moment (D)	7.863	4.569

[1] The calculations were carried out at the B3LYP/6-311+g(d,p) (MeCN) level of theory. Distances between atoms (r) are in angstrom. Bond angles (∠) and dihedral angles (φ) are in degrees. See Figure 8 for the atom numbering.

Table S2. Energies of selected excited singlet and triplet states relative to the singlet ground state of *trans*-DMB.

singlet state	E_{rel} (eV)	triplet state	E_{rel} (eV)
S_1	3.3155	T_1	3.1067
S_2	3.5437	T_2	3.2566
S_3	4.1543	T_3	3.2911
S_4	4.4664	T_4	3.6522
S_5	4.8133	T_5	3.7860
S_6	4.8444	T_6	4.0497
S_7	4.9273	T_7	4.1430

[1]The calculations were performed at the TDDFT/B3LYP/6-311+g(d,p) (MeCN) level of theory.

Synthesis of photoproduct 3,5-dimethoxybenzaldehyde

DMB (140 mg, 0.51 mmol) was dissolved in MeCN (100 mL). The solution was bubbled with high pure nitrogen for 20 min, and then degassed for 10 min in an ultrasonic bath. The solution was irradiated for 60 min with a 300 W high-pressure Hg lamp in a Pyrex photochemical reactor. The reaction mixture was concentrated *in vacuo*. The residue was dissolved with dichloromethane and subjected to column chromatography (sillica gel-*H*, petroleum ether / EtOAc) to yield photoproduct 3,5-dimethoxybenzaldehyde as white powder (43 mg, 51%). M.p. 44-47 °C; ^1H NMR (300 MHz, $CDCl_3$): δ = 3.81 (s, 6H, OCH_3), 6.67 (m, 1H, Ar-H), 6.97 (m, 2H, Ar-H), 9.87 (s, 1H, CHO); ^{13}C NMR (75 MHz, $CDCl_3$): δ = 55.8 (2C, OCH_3), 107.29 (2C, Ar-CH), 107.34 (2C, Ar-CH), 136.6 (Ar-C), 161.4 (Ar-OC), 192.1 (CHO); HRMS (ESI-TOF) calcd for $C_9H_{11}O_3$ $[M + H]^+$ = 167.0705, found 167.0703.

Measurements of quantum yield of photolysis reaction of DMB

To measure the quantum yields of photolysis of DMB in MeCN, [Φ = (rate of photolysis)/(rate of photons absorbed)], the sample solution (~5×10^{-5} M, 3 mL) were prepared and placed in quartz cuvettes with a Teflon stopper, then irradiated with 320 nm wavelength light from a fluorescence spectrometer operated with a 10 nm slit. The absorbances at 270 nm

(A_{270}) and the 320 nm wavelength (A_{ex}) were recorded at certain time intervals after irradiation. The extent of the photolysis was measured by monitoring the increase in the absorbance at 270 nm (A_{270}) due to the generation of photoproduct aryl aldehydes. The A_{270} change (ΔA_{270}) of the solution depends on the extent of the photolysis. The plot of ΔA_{2700} against the irradiation time (t, min) is well fitted as a straight line, where the slope of the straight line B reflected a photoreacting rate of DMB. The intensity of the excitation light beam (I_0, unit: einstein min^{-1}) was measured through ferrioxalate actinometry. The intensity of light absorbed (I_a) by the solution was calculated in terms of Beer's law, $I_a = I_0(1-10^{-A_{ex}})$. The change in the mole extinction coefficients ($\Delta\varepsilon_{270}$) was obtained from the UV absorption spectra of DMB and photoproduct aryl aldehydes. These values allowed the calculation of the quantum yield, $\Phi = BV_0/\Delta\varepsilon_{270}I_a$, wherein V_0 was the volume of irradiation solution, 3×10^{-3} L, the experimental error was within 10 %. To limit the competition of the absorption of the irradiated light between test compounds and the photoproduct, the extent of photolysis of test compounds was controlled within 10% in all of the measurements of the quantum yield.

Cartesian coordinates and electronic energies of the optimized geometries obtained from the DFT or TD-DFT calculations

DMB (S_1)

Electronic energy = -920.360927569 Hartree

C	-1.756046000000	-1.346106000000	-0.279141000000
C	-0.693467000000	-1.095688000000	-1.504298000000
H	-1.203273000000	-0.360576000000	-2.123870000000
O	-1.530469000000	-2.413378000000	0.326417000000
C	-2.687259000000	-0.311267000000	0.098511000000
C	-2.821317000000	0.927453000000	-0.565391000000
C	-3.519923000000	-0.570056000000	1.218060000000
C	-3.756628000000	1.854505000000	-0.127204000000
H	-2.203407000000	1.182069000000	-1.416687000000
C	-4.443959000000	0.372154000000	1.637102000000
H	-3.424505000000	-1.514899000000	1.739967000000
C	-4.575936000000	1.591723000000	0.971007000000
H	-3.842412000000	2.799254000000	-0.652584000000
H	-5.068545000000	0.151551000000	2.496223000000
H	-5.300804000000	2.324792000000	1.303353000000
O	-0.538453000000	-2.234006000000	-2.262117000000
H	0.066630000000	-2.848877000000	-1.825969000000
C	0.548465000000	-0.543450000000	-0.870944000000
C	0.812531000000	0.816619000000	-1.007096000000
C	1.414062000000	-1.375135000000	-0.162483000000
C	1.971355000000	1.347400000000	-0.435110000000
H	0.145757000000	1.467488000000	-1.559953000000
C	2.565670000000	-0.832993000000	0.401217000000
H	1.212024000000	-2.431803000000	-0.019466000000
C	2.856259000000	0.532487000000	0.272838000000

H	3.748475000000	0.946971000000	0.717875000000
O	3.361099000000	-1.697182000000	1.071079000000
O	2.163995000000	2.673333000000	-0.618790000000
C	4.553489000000	-1.197211000000	1.664809000000
H	4.328095000000	-0.438576000000	2.418735000000
H	5.029018000000	-2.051963000000	2.140947000000
H	5.224011000000	-0.781411000000	0.908076000000
C	3.329709000000	3.266180000000	-0.059190000000
H	3.285425000000	4.319768000000	-0.326414000000
H	3.336359000000	3.165829000000	1.029472000000
H	4.235757000000	2.820554000000	-0.478558000000

DMB (Tnπ*)

Electronic energy = -920.360927569 Hartree

C	5.240017000000	0.968581000000	0.784141000000
C	3.883167000000	0.836988000000	1.027756000000
C	3.072693000000	0.047963000000	0.172655000000
C	3.688625000000	-0.610695000000	-0.919215000000
C	5.046379000000	-0.461511000000	-1.146878000000
C	5.834826000000	0.326168000000	-0.303569000000
H	5.841915000000	1.579792000000	1.447503000000
H	3.434273000000	1.348564000000	1.872148000000
H	3.105406000000	-1.244582000000	-1.575860000000
H	5.499805000000	-0.971748000000	-1.989397000000
H	6.896660000000	0.433338000000	-0.489721000000
C	1.673442000000	-0.074281000000	0.420962000000
O	1.157519000000	0.431341000000	1.516168000000
C	0.718253000000	-0.940978000000	-0.400011000000
H	1.013422000000	-0.819127000000	-1.445131000000
C	-0.730447000000	-0.491709000000	-0.256862000000
C	-1.036565000000	0.861646000000	-0.387560000000
C	-1.735324000000	-1.424450000000	-0.043957000000
C	-2.362839000000	1.277895000000	-0.298338000000
H	-0.263014000000	1.601776000000	-0.559565000000
C	-3.062653000000	-0.993418000000	0.049160000000
H	-1.524753000000	-2.482674000000	0.042463000000
C	-3.392684000000	0.356332000000	-0.075554000000
H	-4.419252000000	0.682771000000	-0.002920000000
O	0.914431000000	-2.309484000000	-0.113965000000
H	0.645215000000	-2.480315000000	0.798772000000
O	-3.980234000000	-1.965864000000	0.262883000000
O	-2.576310000000	2.607887000000	-0.437850000000
C	-5.346486000000	-1.586873000000	0.368048000000
C	-3.911284000000	3.088497000000	-0.346057000000

H	-5.696213000000	-1.116941000000	-0.555215000000
H	-5.501563000000	-0.907794000000	1.210823000000
H	-5.899476000000	-2.508333000000	0.537890000000
H	-4.540700000000	2.653267000000	-1.126975000000
H	-3.850335000000	4.165483000000	-0.488101000000
H	-4.339364000000	2.874549000000	0.637080000000

DMB (Tππ*)

Electronic energy = -920.366440043 Hartree

C	-1.792236000000	-1.355150000000	-0.394137000000
C	-0.798554000000	-1.193953000000	-1.557296000000
H	-1.173299000000	-0.538763000000	-2.346330000000
O	-1.776806000000	-2.505986000000	0.154027000000
C	-2.573321000000	-0.258058000000	0.074649000000
C	-2.605273000000	1.027212000000	-0.548285000000
C	-3.392109000000	-0.428270000000	1.234046000000
C	-3.397618000000	2.045620000000	-0.046920000000
H	-2.021084000000	1.219388000000	-1.440354000000
C	-4.173572000000	0.602663000000	1.720592000000
H	-3.388183000000	-1.394203000000	1.725377000000
C	-4.191713000000	1.857816000000	1.093452000000
H	-3.401585000000	3.006678000000	-0.551773000000
H	-4.782560000000	0.435693000000	2.603953000000
H	-4.805837000000	2.662482000000	1.479449000000
O	-0.536851000000	-2.470748000000	-2.094474000000
H	-0.872218000000	-3.074051000000	-1.404160000000
C	0.464455000000	-0.593691000000	-0.971879000000
C	0.825247000000	0.722551000000	-1.175859000000
C	1.280158000000	-1.376131000000	-0.152322000000
C	1.991190000000	1.276099000000	-0.530539000000
H	0.259402000000	1.375442000000	-1.830814000000
C	2.442361000000	-0.815748000000	0.484735000000
H	1.063584000000	-2.423066000000	0.026050000000
C	2.804368000000	0.514002000000	0.304915000000
H	3.677324000000	0.932416000000	0.781488000000
O	3.103387000000	-1.674854000000	1.227197000000
O	2.201180000000	2.538553000000	-0.816189000000
C	4.283994000000	-1.240310000000	1.920280000000
H	4.031175000000	-0.440858000000	2.618143000000
H	4.641361000000	-2.113605000000	2.457936000000
H	5.033191000000	-0.902571000000	1.202332000000
C	3.334553000000	3.213819000000	-0.244640000000
H	3.287667000000	4.229237000000	-0.626517000000
H	3.256148000000	3.212081000000	0.843418000000

H	4.256047000000	2.727151000000	-0.567427000000

cis-DMB (S_0)

Electronic energy = -920.331914677 Hartree

C	2.008450337741	-1.142606077839	0.750801226514
C	0.824997285015	-0.915508730078	1.714116633616
H	1.086103759172	-0.116469405792	2.413492266903
O	2.401795634622	-2.292470440785	0.607400806750
C	2.624051269850	-0.008613346749	0.016886779349
C	2.375219739331	1.330415967815	0.353346054420
C	3.504088418120	-0.297869725771	-1.038926162769
C	2.996215982751	2.357547695764	-0.352272245301
H	1.709454386257	1.582472367991	1.167822717864
C	4.114870619866	0.729101657239	-1.747122582826
H	3.693933767217	-1.332738976561	-1.294621201585
C	3.862617778511	2.059616078481	-1.403618485448
H	2.804395077397	3.388810996252	-0.080756219114
H	4.786723834392	0.497135575040	-2.565145062986
H	4.341520601180	2.861377052906	-1.953981768953
O	0.613910835228	-2.101411714714	2.465827611073
H	1.135469477040	-2.792265493458	2.021879231747
C	-0.433370927293	-0.510354100451	0.951246281664
C	-1.140254631107	0.624727546287	1.339567395114
C	-0.891506641800	-1.299027967460	-0.105034675503
C	-2.313887625204	0.975465015108	0.663515799422
H	-0.805595170517	1.242591330273	2.164246136592
C	-2.065137371019	-0.944585649342	-0.773226752890
H	-0.361580585296	-2.190080993230	-0.419561302957
C	-2.786571620344	0.196182877963	-0.397545389406
H	-3.692444323765	0.468806482185	-0.915455441929
O	-2.440280997282	-1.769891063685	-1.791990463415
O	-2.942466645221	2.099181491907	1.111895138607
C	-3.625364720285	-1.465727331232	-2.531455004012
H	-3.542401491252	-0.495683053343	-3.029702102105
H	-3.714659999611	-2.250385519224	-3.279898174370
H	-4.508393164666	-1.476737731748	-1.886179161181
C	-4.155415540380	2.514313270133	0.478697077495
H	-4.467867956004	3.414930317412	1.003119474176
H	-3.990471579253	2.746646996109	-0.577226659628
H	-4.931871354010	1.749860747000	0.573046831416

trans-DMB (S_0)

Electronic energy = -920.387085519 Hartree

C	-1.887046144019	0.930621069207	1.075144080295

C	-0.765870056689	0.222670016999	1.877448144039
O	-2.100181160648	2.108289161539	1.321381102388
C	-2.678614206818	0.202377015669	0.040797003092
C	-2.304954176598	-1.053349079195	-0.461967035050
C	-3.829651290049	0.826798064610	-0.467432035829
C	-3.064112233729	-1.663390125869	-1.457586113885
H	-1.425903107797	-1.553008117440	-0.082296006373
C	-4.592075349784	0.209970015970	-1.451654111008
H	-4.113828315314	1.796379136720	-0.077805006064
C	-4.208108322615	-1.036917077842	-1.951283150195
H	-2.763513208562	-2.629515199575	-1.846046141654
H	-5.482171416709	0.697545055721	-1.832087139048
H	-4.799793364127	-1.517741115556	-2.722082207203
C	0.524497040307	0.135977010187	1.068526083783
C	1.196373089889	-1.075986080675	0.934218072181
C	1.040871079663	1.296336101004	0.487679037368
C	2.391166183515	-1.124895086826	0.208564015832
H	0.813796063183	-1.979889149333	1.386369106333
C	2.233999169993	1.235780094106	-0.234431017871
H	0.538814042964	2.250610170842	0.587735045564
C	2.921976224792	0.025064001917	-0.384070029405
H	3.843104293370	-0.019112001485	-0.943136074104
O	2.664999201174	2.416549186284	-0.765944056836
O	2.981199228909	-2.352989177729	0.129146009779
C	3.881089297775	2.435567186892	-1.516688116331
H	3.812804292022	1.790612136601	-2.397470182123
H	4.015338306114	3.467723262317	-1.833837138794
H	4.731762363455	2.131043164109	-0.900200066190
C	4.210644319912	-2.485284188493	-0.587790042955
H	4.484963344539	-3.535460267985	-0.511293039223
H	4.085517311325	-2.217803171110	-1.640951126640
H	4.997076384128	-1.870182143527	-0.141040010886
O	-1.143766087286	-1.072422082486	2.328637176287
H	-1.789898138906	-0.974729072433	3.036707234108
H	-0.587195046640	0.880062067226	2.734829208697

Alcohol radical of DMB

Electronic energy = -575.357228886 Hartree

C	2.912657000000	-0.906822000000	0.000248000000
H	3.202904000000	-1.947960000000	0.001544000000
O	3.983070000000	-0.070729000000	0.000301000000
H	3.703281000000	0.853442000000	-0.002663000000
C	1.560390000000	-0.501775000000	-0.000111000000

C	0.552569000000	-1.502493000000	-0.000033000000
C	1.163976000000	0.860005000000	-0.000413000000
C	-0.788387000000	-1.146042000000	-0.000085000000
H	0.820680000000	-2.552074000000	0.000156000000
C	-0.186491000000	1.192439000000	-0.000151000000
H	1.880096000000	1.673686000000	-0.000727000000
C	-1.182858000000	0.203405000000	-0.000097000000
H	-2.226573000000	0.472453000000	-0.000135000000
O	-0.464872000000	2.530493000000	0.000115000000
O	-1.680293000000	-2.183079000000	-0.000075000000
C	-1.829022000000	2.956359000000	0.000272000000
H	-2.353185000000	2.607930000000	0.894773000000
H	-1.797585000000	4.044095000000	0.000564000000
H	-2.353273000000	2.608485000000	-0.894382000000
C	-3.079488000000	-1.894878000000	0.000028000000
H	-3.579323000000	-2.861588000000	-0.000092000000
H	-3.370112000000	-1.336709000000	0.894798000000
H	-3.370218000000	-1.336426000000	-0.894515000000

Benzoyl radical of DMB

Electronic energy = -345.021579144 Hartree

C	-2.009343000000	-0.504866000000	0.001670000000
O	-2.933066000000	0.246410000000	-0.001128000000
C	-0.564108000000	-0.227261000000	0.000089000000
C	0.333871000000	-1.302127000000	0.000031000000
C	-0.087334000000	1.095678000000	0.000130000000
C	1.704328000000	-1.058942000000	-0.000471000000
H	-0.050950000000	-2.315197000000	0.000062000000
C	1.281405000000	1.330136000000	0.000124000000
H	-0.791550000000	1.919137000000	0.000329000000
C	2.175360000000	0.254352000000	-0.000119000000
H	2.402479000000	-1.887318000000	-0.000730000000
H	1.656761000000	2.346792000000	0.000443000000
H	3.242716000000	0.443486000000	0.000204000000
